# Nuances of Whitefly Vector–Crinivirus Interactions Revealed in the Foregut Retention and Transmission of Lettuce Chlorosis Virus by Two *Bemisia tabaci* Cryptic Species

**DOI:** 10.3390/v13081578

**Published:** 2021-08-10

**Authors:** Angel Y. S. Chen, Jaclyn S. Zhou, Jin-Xiang Liu, James C. K. Ng

**Affiliations:** 1 Department of Microbiology and Plant Pathology, University of California, Riverside, CA 92521, USA; yun-shu.chen@ucr.edu (A.Y.S.C.); jzhou003@ucr.edu (J.S.Z.); 2 Citrus Research Institute, Southwest University, Beibei, Chongqing 400712, China; ljinxiang@126.com; 3 Chinese Academy of Agricultural Sciences, No. 12 Zhongguancun South St., Beijing 100080, China; 4 Center for Infectious Diseases and Vector Research, University of California, Riverside, CA 92521, USA

**Keywords:** crinivirus, transmission, whitefly vector, retention

## Abstract

Lettuce infectious yellows virus is the first crinivirus for which the retention of purified virions ingested into the whitefly (*Bemisia tabaci* New World (NW)) vector’s foregut, has been demonstrated to be a requisite for successful virus transmission. This key finding supports the hypothesis that the determinant of foregut retention and transmission is present on the virion itself. However, whether this is also true for other criniviruses has not been established. Here, we provide evidence that lettuce chlorosis virus (LCV) acquired from plants is retained in the foreguts of both the *B. tabaci* NW and Middle East–Asia Minor 1 (MEAM1) vector species and transmitted upon inoculation feeding. An association between foregut retention and transmission by NW vectors is also observed following the acquisition and inoculation feeding of LCV virions purified using a standard procedure involving 2% or 4% (*v*/*v*) Triton™ X-100 (TX-100). However, while virions purified with 2% or 4% TX-100 are also retained in the foreguts of MEAM1 vectors, transmission is observed with the 4% TX-100-purified virions or when more vectors are used for acquisition and inoculation feeding. These results suggest that an intrinsic difference exists between NW and MEAM1 vectors in their interactions with, and transmission of, LCV virions.

## 1. Introduction

Successful insect vector transmission of plant viruses requires a confluence of direct and indirect contributions from an insect, virus, and a plant [1,2,3]. The complexities in the interactions among the three participants present a significant challenge for understanding the biological and molecular basis underlying pathogenesis and disease transmission. For emerging viruses in the genus *Crinivirus* (family Closteroviridae) [4], complexity is exemplified by their relatively large genome sizes and complicated biology associated with, but not limited to, phloem tropism and low titer infections. Criniviruses form filamentous, rod-shaped particles that separately encapsidate each of their bipartite, plus-sense, single-stranded RNA genomes [4,5]. Open reading frames (ORFs) located in RNA 1 encode for proteins that are essential for viral replication. RNA 2 contains between 7 and 10 ORFs (depending on the crinivirus), the most conspicuous of which are those that make up the hallmark gene array of viruses in the family Closteroviridae: encoding for a hydrophobic protein, a heat shock protein 70 homolog (Hsp70 h), a 50–60 kDa protein (depending on the virus), a major coat protein (CP), and a minor coat protein (CPm) [6,7]. Proteins encoded by the ORFs on RNA 2 are associated with, or have been implicated in, different processes in the viral infection cycle, including virion assembly, long-distance movement, suppression of RNA silencing, and vector transmission [8,9,10,11,12,13].

Another factor that contributes to the difficulty in understanding criniviruses is their complex transmission mechanisms involving different but specific whitefly vectors in the family Aleyrodidae (order Hemiptera). Crinivirus transmission by whitefly vectors is achieved in a non-circulative, semipersistent (NCSP) manner [1,14], a mode of transmission that exhibits a number of characteristics, including the following: virus acquisition and inoculation are accomplished in minutes to hours, virus retention within the vector persists for hours to days, and internalization and circulation of the virus within the vector are not a prerequisite for transmission [15]. Our current knowledge about the NCSP transmission mechanism of criniviruses has been primarily gathered from studies of lettuce infectious yellows virus (LIYV) and its specific whitefly vector, the New World (NW) species of *Bemisia tabaci* (Gennadius) [2,10,16,17]. NW vectors consistently transmit LIYV to target plants following the acquisition access feeding of a sugar-based artificial diet containing highly purified LIYV virions sandwiched between a pair of stretched parafilm™ membranes, i.e., membrane feeding. The transmissibility of purified virions acquired in vitro (by membrane feeding) from a plant-free environment provides clear evidence that the transmission of LIYV is facilitated by determinant(s) located solely on the virion capsid, and not on other viral-encoded non-capsid proteins [17]. Using a combination of membrane feeding, virus transmission, and immunofluorescent localization approaches, also referred to as a virus or virion retention and transmission (VRT) assay [2], our studies have shown that purified LIYV virions are retained in the foreguts of a significant number of NW vectors, and this is consistently associated with virus transmission when the virion-fed vectors are allowed inoculation access feeding on plants. In contrast, with non-vector whiteflies *B. tabaci* Middle East–Asia Minor 1 (MEAM1), the number of insects that retain purified LIYV virions in their foreguts is significantly reduced, and this is consistent with the absence of virus transmission following inoculation access feeding [10,18]. These observations are further backed by biochemical and molecular data supporting the role of the LIYV CPm in mediating the binding of virions to the vector’s foregut [10,16,17]. The mechanism underlying the inoculation of LIYV virions retained in the foregut by NW vectors is not understood but may be influenced by the physical properties in the foregut environment such as the dietary pH [19].

In contrast to LIYV, knowledge about the whitefly retention and transmission of other criniviruses has been considerably limited. In one study, cucurbit chlorotic yellows virus (CCYV) was found localized in the foregut of whitefly vectors, *B. tabaci* Mediterranean (MED), following their acquisition access feeding of virus-infected plants [20]. In our previous study, virions of a wild-type (WT) population of greenhouse-maintained lettuce chlorosis virus (from here on referred to as GH-LCV) purified from *Chenopodium murale* plants were transmissible by both the NW and MEAM1 vectors [21]. In addition, both vectors also transmitted the virions of a clonal population of WT LCV purified from *Nicotiana benthamiana* plants infiltrated with *Agrobacterium* harboring the infectious clone of the virus [22]. No foregut retention of purified virions has hitherto been reported for CCYV, LCV, or any other criniviruses, other than LIYV. 

In this study, we have expanded our understanding of LCV transmission by illuminating the foregut retention sites of LCV in both the NW and MEAM1 vectors. Using VRT assays, we examined the whitefly transmission and foregut retention of both in planta acquired LCV and LCV virions acquired by membrane feeding. Our results were consistent with in planta acquired LCV being retained in the foreguts of, and transmitted by, both vector species. In the case of purified LCV virions acquired by membrane feeding, foregut retention was consistently observed in both vector species. However, nuances associated with the LCV virion purification procedure were observed, which resulted in differential virus transmission by the two vector species. The possible implications of these nuances and their effects on the retention and transmission of criniviruses are discussed. This is also the first time that an association between foregut retention and transmission has been demonstrated for a crinivirus that is transmitted by two *B. tabaci* vector species.

## 2. Materials and Methods

### 2.1. Maintenance of Viruses and Whitefly Colonies

GH-LCV was maintained by whitefly-mediated plant-to-plant transmission in *Lactuca sativa* (lettuce) and *Chenopodium murale* plants grown under natural light conditions in a greenhouse [7,21]. Colonies of non-viruliferous *B. tabaci* NW and MEAM1 were reared and maintained on lima bean (*Phaseolus lunatus*) and mustard (*Brassica napus*) plants, respectively [10].

### 2.2. Virion Purification and Quantification

Virions of GH-LCV were purified from infected *C. murale* plants. The standard virion purification procedure was similar to the method described in Ng and Chen [21]. Briefly, virus-infected tissues were grounded in liquid nitrogen and stirred in an extraction buffer (0.5% (*w*/*v*) sodium sulfite (Na_2_SO_3_), 0.5% (*v*/*v)* β-mercaptoethanol, in 10 mM Tris-HCl, pH 7.4) on ice for 10 min. Tissue debris was removed by passing the tissue/buffer mixture through cheesecloth. The filtrate was added with 2% (*v*/*v*) Triton X-100 (TX-100) and stirred on ice for 2 h. A modification in the purification procedure involved using 4% (*v*/*v*) TX-100 at this step. Following the stirring, the solution was centrifuged at 10,000× *g* for 10 min (at 4 °C). The supernatant was subjected to one round of ultracentrifugation in a 20% sucrose cushion (20% (*w*/*v*) sucrose in 10 mM Tris-HCl, pH 7.4) at 91,075.7× *g* for 1 h (4 °C). The pellet was soaked in 2.5–5 mL of 10 mM Tris-HCl, pH 7.4, and kept on ice overnight. The next day, the pellet was resuspended and stirred on ice for 2 h with 2% (*v*/*v*) TX-100. The solution was centrifuged at 6000× *g* for 10 min at 4 °C, and the supernatant was subjected to a second round of ultracentrifugation in a 20% sucrose cushion (20% (*w*/*v*) sucrose in 1× TE (10 mM Tris, pH 7.4, 1 mM EDTA) at 92,387× *g* for 2 h (4 °C). The pellet was resuspended in a 1× artificial diet (15% sucrose and 1% bovine serum albumin in 1× TE, pH 7.4) and used immediately for whitefly feeding. Purified virions were quantified using the methods as previously described [22].

### 2.3. VRT Assays

VRT assays were essentially carried out according to a previously described method [10] with minor modifications. For VRT assay experiments of in planta acquired LCV, virus acquisition was performed by releasing thousands of whiteflies into two separate insect-proof tents, one containing a lettuce plant infected with GH-LCV and the other containing an uninfected lettuce plant. Following a 12–16 h acquisition access period (AAP), two different sampling approaches were used to prepare the whiteflies for VRT assays. In the first approach, whiteflies (approximately 100 per cage) were randomly sampled and used directly for either VR or transmission assays (see Table 1). The second approach was performed to mimic the method used for sampling whiteflies to determine the foregut retention and transmission of purified virions [10]. In addition, it allowed us to more accurately perform the correlation analysis aimed at assessing the degree of association between foregut retention and virus transmission. In this approach, whiteflies (approximately 200 per cage) were randomly sampled, pooled, and then redistributed into two groups of approximately equal numbers. One group was used for the VR assay, while the other group was used for the transmission assay by transferring the whiteflies to an uninfected lettuce seedling for an overnight inoculation access period (IAP) (Table 1). VRT procedures involving purified virions and membrane feeding were performed, as previously published [10], and are briefly described in the relevant Results section.

To assay for the retention of LCV by immunofluorescent localization, viruliferous whiteflies were membrane fed on an artificial diet containing the following in sequential order: (1) polyclonal IgG against LCV virions (a gift from Dr. Rebecca Creamer, New Mexico State University) [23] at 1:800 dilution and (2) goat anti-rabbit IgG conjugated with Alexa Fluor 488 (Thermo Fisher Scientific, Waltham, MA, USA) at 1:200 dilution. To visualize the retention of virus in the whitefly’s foregut, the heads of whiteflies were dissected and observed under a Nikon Labophot fluorescence microscope fitted with FITC/CY3, double bandpass filters. The images of the whiteflies’ heads were taken using a Canon EOS T5i digital single-lens reflex camera. The transmissibility of LCV was determined by symptom development (interveinal yellowing) of target lettuce plants 4 to 5 weeks post-inoculation, and/or the detection of viral sequence by RT-PCR using specific primers to LCV sequences [22]. 

### 2.4. Statistical Analysis

The association between LCV retention in the vectors’ foreguts and transmission was analyzed by point biserial correlation (PBC) analysis. In the analysis, the percentage of whiteflies harboring fluorescent signals in their foreguts was the continuous variable, while transmission (either positive or negative) was the dichotomous variable. Pairwise retention and transmission datasets were analyzed after the normality requirement for the continuous variable was met. The PBC analysis provided the correlation coefficients (rpb) and two-tailed *p*-values. A positive rpb value indicates a positive correlation, where vectors retaining LCV in their foreguts and virus transmissibility increase together. All statistical analyses were performed using the GraphPad Prism Software (GraphPad, La Jolla, CA, USA).

## 3. Results

### 3.1. In Planta Acquired GH-LCV Is Retained in the Foreguts of B. tabaci NW and MEAM1 Vectors and Transmitted

**Figure 1:**
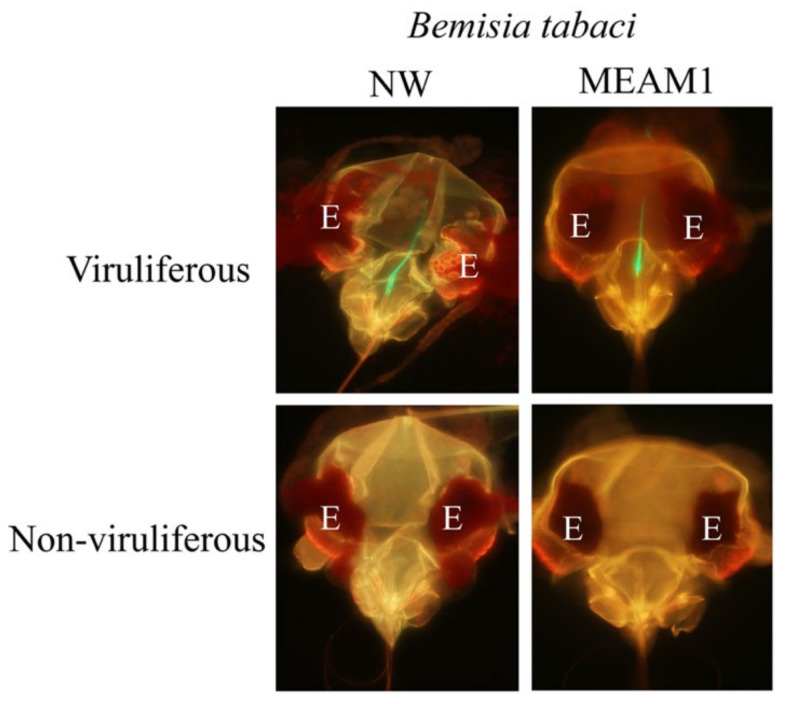
Foregut retention of in planta acquired LCV (GH-LCV) in two *B. tabaci* vector species. Representative images of the dissected heads of viruliferous (top panels) and non-viruliferous (bottom panels) vectors that were given acquisition access feeding on infected and uninfected source plants, respectively. Vectors were subjected to immunofluorescent localization (virus retention assay) and examined by wide-field fluorescence microscopy. E, eye; NW, *B. tabaci* New World vector; MEAM1, *B. tabaci* Middle East–Asia Minor 1 vector.

To determine whether in planta acquired GH-LCV is retained in the foregut of whitefly vectors, three repeated experiments were conducted. In these experiments, NW or MEAM1 vectors were given an overnight AAP on GH-LCV, infected or uninfected (negative control), lettuce plants, and randomly collected (by either of two sampling approaches) for VRT assays (Table 1). In the first experiment, results from the retention assays showed that fluorescent signals were observed in the foreguts of both NW and MEAM1 vectors. The average percentages of sampled NW and MEAM1 vectors with fluorescent signals in their foreguts were 5.7% (23 of 419 foreguts examined) and 26.4% (164 of 603 foreguts examined), respectively (Table 1 and Figure 1). These percentages were based on a cumulative total determined from four and six independent samplings of NW and MEAM1 vectors, respectively. No fluorescent signals were seen in the foreguts of whiteflies that fed on uninfected plants (data not shown). Representative images of a NW and MEAM1 vector with no fluorescent signals in their foreguts are shown in Figure 1. NW and MEAM1 vectors that were randomly sampled from the infected source plants (from which whiteflies were collected for VR assays) both transmitted the virus to target lettuce plants, with two out of four and three out of six target plants developing infection, respectively, after they were exposed for an overnight IAP to each vector species (Table 1). 

In Experiments 2 and 3, results from the retention assays were consistent with those obtained from Experiment 1, i.e., retention of GH-LCV was also observed in the foreguts of both NW and MEAM1 vectors. The average percentages of sampled whiteflies that showed fluorescent signals in their foreguts were 23.8% (153 of 667 foreguts examined) and 18.2% (65 of 347 foreguts examined) for NW vectors, and 27.5% (156 of 587 foreguts examined) and 19.6% (105 of 531 foreguts examined) for MEAM1 vectors (Table 1). These percentages were based on a cumulative total determined from four to six independent samplings. No fluorescent signals were observed in the foreguts of both vectors that fed on uninfected plants (data not shown). Transmission was observed when subsets of insects from pooled whitefly samples were transferred to uninfected target lettuce plants for inoculation feeding. The results of the transmission assays (combined from Experiments 2 and 3) showed that a cumulative total of 7 of 10 and 8 of 11 target plants given access to viruliferous NW and MEAM1 vectors, respectively, were infected with GH-LCV (Table 1). In contrast, no transmission was observed in target plants exposed to NW or MEAM1 vectors that had acquisition fed on uninfected source plants (data not shown). The raw data of retention and transmission for Experiments 2 and 3—13 and 15 pairs of datasets (not shown) for viruliferous NW and MEAM1 vectors, respectively, from which results shown in Table 1 were generated, together with those of non-viruliferous vectors fed on uninfected plants (negative controls), were analyzed for a correlation between percentage of foregut retention and virus transmission. The PBC coefficient (rpb) values of 0.6451 (*p* = 0.0173) and 0.572 (*p* = 0.0259) were obtained for NW and MEAM1 vectors, respectively, indicating a moderate to strong positive correlation in the association of foregut retention and virus transmission.

### 3.2. Purified GH-LCV Virions Are Retained in the Foreguts of B. tabaci NW Vectors and Transmitted upon Inoculation Access Feeding

**Figure 2:**
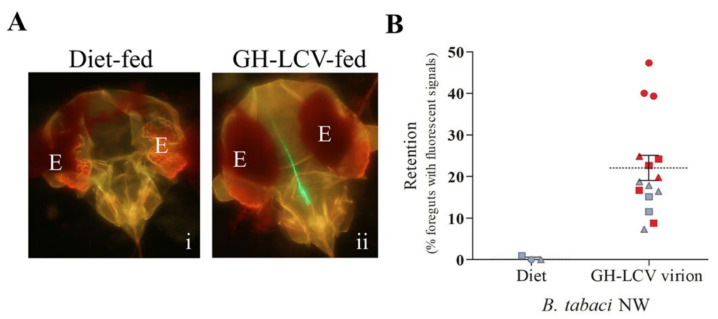
Foregut retention and transmission of purified GH-LCV virions by *B. tabaci* NW vectors. GH-LCV virions were purified from *C. murale* plants using an extraction buffer with 2% or 4% Triton™ X (TX)-100. Whiteflies allowed acquisition accessed feeding on an artificial diet containing GH-LCV virions, or an artificial diet alone, were used in VRT assays. (**A**) Representative images of the dissected heads of whiteflies that fed on artificial diet alone (**i**) or artificial diet containing purified GH-LCV virions (**ii**). The eye of the whitefly is labeled E in each image; (**B**) the foregut retention and transmission results of three repeated experiments for whiteflies fed on purified GH-LCV virions or diet control. Each data point represents the percentage of foreguts with fluorescent signals for one independent cage of whiteflies (i.e., one technical replicate of approximately 100 foreguts). The three symbols (circle, triangle, or square) represent each of the three repeated experiments, i.e., circle and triangle (Experiments 1 and 2, respectively, 2% (*v*/*v*) TX-100) and square (Experiment 3, 4% (*v*/*v*) TX-100). Technical replicates within an experiment share the same symbol. The mean (dashed line) and standard error (solid line) (22.02% ± 3.02%) are shown. Transmission results are presented by color symbols, indicating that the target plants were infected (red) or not infected (gray) by GH-LCV.

Our previous study showed that both NW and MEAM1 vectors transmitted purified virions of GH-LCV [21]; however, virion retention in the vectors was not investigated because the VRT assay that was developed using LIYV [10,18] had not yet been established. A demonstration of the binding of purified virions to a vector’s foregut and its association with transmission would lend support to the hypothesis that, as with LIYV, the determinant mediating LCV retention and transmission by whitefly vectors also resides in the virion capsid. Here, we tested this hypothesis by first using *B. tabaci* NW vectors. Three VRT assay experiments were performed with virions of GH-LCV purified from infected *C. murale* plants using a standard procedure in which the extraction buffer contained the non-ionic detergent Triton™ X-100 (TX-100) at a percent volume (*v*/*v*) concentration of 2% (Experiments 1 and 2) [17,21] or, with a slight modification, 4% (Experiment 3). The rationale for using 4% (*v*/*v*) TX-100 in the purification procedure is explained below (Results Section 3.3). In each experiment, technical replicates of membrane feeding (with approximately 200 whiteflies per membrane feeding cage) were performed in which whiteflies were allowed to acquire the artificial diet augmented with GH-LCV virions or the artificial diet alone. Following an overnight AAP, whiteflies were split equally into two groups where one group, the VR group, was used for the VR assay and the other group, the transmission group, for the overnight IAP on a lettuce seedling. In all three experiments, with a cumulative total of 15 technical replicates and virion concentrations ranging from 118.13 to 960 ng µL^−1^, fluorescent signals were observed in the foreguts of 22.02% ± 3.02% (mean ± S.E.) (319 of 1467 foreguts examined) of NW vectors from the VR groups (Figure 2). In contrast, no fluorescent signals were observed within the foreguts of NW vectors that fed on the diet alone controls (Figure 2). The NW vectors from the transmission groups given access to target plants for inoculation feeding transmitted the virus to 9 of 15 plants (Figure 2B) based on symptom development and RT-PCR results. The 18 pairs of datasets (including the diet controls) in Figure 2B were analyzed for a correlation between the two variables, i.e., percentage of foregut retention and virus transmission. The rpb value of 0.6598 (*p* = 0.0029) was obtained, indicating a moderate to strong positive correlation in the association of foregut retention and virus transmission (i.e., virus transmission (Figure 2B, red-filled symbols)) was more frequently observed as the percentage of vectors retaining virions in their foreguts increased above the mean). 

### 3.3. Purified GH-LCV Virions Are Retained in the Foreguts of B. tabaci MEAM1 Vectors, and Transmission Is Influenced by the TX-100 Concentration Used for Virion Purification

**Figure 3:**
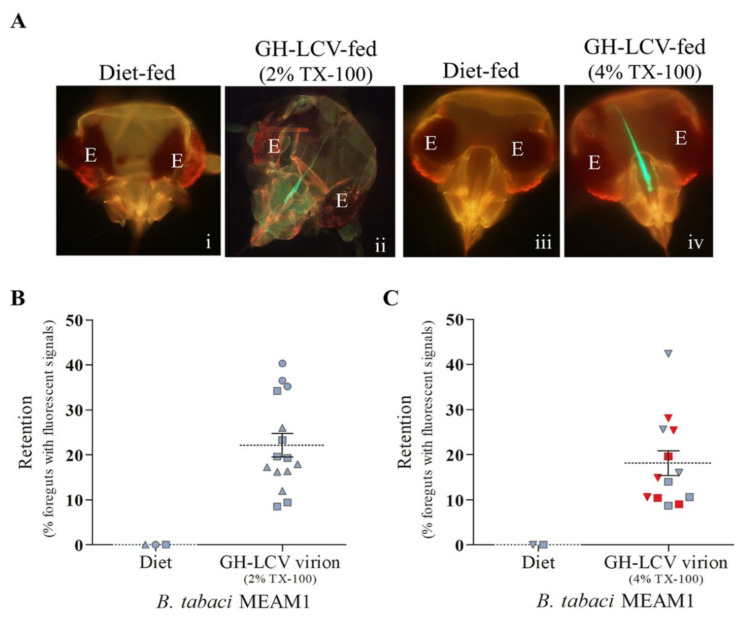
Foregut retention and transmission of purified GH-LCV virions by *B. tabaci* MEAM1 vectors. GH-LCV virions were purified from *C. murale* plants using an extraction buffer with either 2% or 4% Triton™ X (TX)-100. Whiteflies were given acquisition access to an artificial diet containing purified GH-LCV virions or an artificial diet alone, and then subjected to VRT assays. (**A**) Representative images of the dissected heads of whiteflies that fed on an artificial diet alone (**i**, **iii**) and an artificial diet containing either GH-LCV virions purified using TX-100 at a percent volume (*v*/*v*) concentration of 2% (**ii**) or 4% (**iv**). The eye of the whitefly is labeled E in each image; (**B**) the results of three biological repeats (represented by circle, triangle and square) of VRT assays for whiteflies that fed on GH-LCV virions purified using 2% TX-100 or diet alone controls; (**C**) the results of two biological repeats (represented by square (Experiment 1) and inverted triangle (Experiment 2)) of VRT assays for whiteflies that fed on GH-LCV virions purified using 4% TX-100 or diet alone controls. Each data point represents the percentage of foreguts with fluorescent signals for one technical replicate. Technical replicates within the same experiment share the same symbol within the graph. The means (dashed lines) and standard errors (solid lines) are indicated. Transmission results are presented by color symbols, indicating that the target plants were infected (red) or not infected (gray) by GH-LCV.

Using the same approach as we did with *B. tabaci* NW in the preceding section, we performed VRT assays using *B. tabaci* MEAM1 to determine if, similar to the interactions between NW vectors and LIYV [10] or LCV (Figure 2), the interaction between MEAM1 vectors and LCV was also mediated by the virion capsid. In three independent experiments, GH-LCV was purified from infected *C. murale* plants using an extraction buffer containing 2% (*v*/*v*) TX-100. The resulting purified virions, at concentrations of 423.7 ng µL^−1^ (Experiment 1), 134 ng µL^−1^ (Experiment 2), and 504.6 ng µL^−1^ (Experiment 3), were acquired by MEAM1 vectors (via membrane feeding) for VRT assays. The results from the retention assays showed that fluorescent signals were observed in the foreguts of 22.12% ± 2.62% (mean ± S.E.) (311 of 1440 foreguts examined) of the MEAM1 vectors from the VR groups that fed on the purified virions, while no signals were observed for MEAM1 vectors that fed on diet alone controls (Figure 3A,B). None of 15 target lettuce seedlings was infected after being exposed for an overnight IAP to the virion fed MEAM1 vectors from the transmission groups, and no transmission was observed in plants exposed to MEAM1 vectors that fed on the diet alone controls (Figure 3B).

The lack of transmission of LCV virions purified using 2% (*v*/*v*) TX-100 suggested that the quality of the purified virions could be suboptimal, although there was no a priori knowledge of the underlying causes. We sought to improve the quality of the virion preparations by using an extraction buffer with 4% (*v*/*v*) TX-100. In a preliminary set of two independent experiments, GH-LCV virions purified with 4% (*v*/*v*) TX-100 were membrane fed to MEAM1 vectors for VRT assays. The concentrations of the purified GH-LCV virions in these experiments were 753.9 ng µL^−1^ (Experiment 1) and 1049 ng µL^−1^ (Experiment 2). The results from the retention assays showed that fluorescent signals were observed in the foreguts of 24.03% ± 3.38% (mean ± S.E.) (287 of 1184 foreguts examined) of the MEAM1 vectors from the VR groups, whereas no signals were observed in the foreguts of MEAM1 vectors that fed on diet alone controls. Among a total of 12 target lettuce plants tested in the two transmission assays, five plants developed chlorotic symptoms following IAP exposure to virion fed MEAM1 vectors from the transmission groups. To verify that the symptoms coincided with LCV infection, we went a step further by performing a plant-to-plant transmission using a symptomatic plant (from each experiment) as a source for acquisition feeding. The MEAM1 vectors were released onto the symptomatic plant in an insect-proof tent for an overnight AAP, following which they were transferred to target lettuce seedlings for inoculation access feeding. LCV infection of the target plants was confirmed by both symptom development and RT-PCR (data not shown). 

We carried out another set of two independent VRT assays to confirm the above findings, and the results are shown in Figure 3A,C. Artificial diet containing virions purified with 4% (*v*/*v*) TX-100, at concentrations of 194 ng µL^−1^ (Experiment 1) and 1190 ng µL^−1^ (Experiment 2), or the diet alone controls, were given access to MEAM1 vectors for an overnight AAP. In Experiment 1, virion acquisition feeding was performed using six membrane feeding cages (i.e., six technical replicates, with 200 MEAM1 vectors per replicate). Following acquisition feeding, vectors from each membrane feeding cage were separated into the VR and transmission groups for VRT assays. In Experiment 2, virion acquisition feeding was performed using seven membrane feeding cages, of which four cages contained 200 MEAM1 vectors each and three cages contained 300 MEAM1 vectors each (prior to vectors being separated into the two groups for VRT assays). The results of the retention assays showed that fluorescent signals were observed in the foreguts of 18.1% ± 2.75% (mean ± S.E.) (202 of 1173 foreguts examined) of the MEAM1 vectors, while no signals were observed in the foreguts of whiteflies fed on diet controls (Figure 3A,C). Among a total of 13 target lettuce plants tested for infection following exposure to MEAM1 vectors from the transmission group, seven plants were positive for GH-LCV based on symptom development and RT-PCR results (Figure 3C). A PBC analysis of the 15 pairs of datasets (including the diet controls), and shown in Figure 3C, was performed and the rpb value of 0.1015 (*p* = 0.7188) was obtained.

Taken together, these results indicate a very weak positive correlation in the association of foregut retention of purified GH-LCV virions and their transmission by MEAM1 vectors, and transmission can be affected by the TX-100 concentration used in the virion purification procedure.

## 4. Discussion

Retention within the insect vector is a critical stage in the disease cycle of all insect-transmitted viruses because it represents the first contact(s) between the virus and the vector following virus uptake. It is becoming evident that retention in the foregut, an area in the alimentary canal that encompasses the cibarium (sucking pump), anterior pharynx, and posterior esophagus, is an important process associated with the transmission of criniviruses by whitefly vectors in the *B. tabaci* species complex [10,20]. Most of the evidence supporting this association is based on studies of LIYV and its specific (and exclusive) vector *B. tabaci* NW [10,16,17,18,19], although much more remains to be discovered. Studies of retention and transmission of other criniviruses have been more limited, and much of the biological and molecular basis underlying crinivirus–vector interactions and transmission are not well understood. For example, one investigation showed that CCYV acquired from infected plants was localized in the foregut of its whitefly vector *B. tabaci* MED; however, neither the transmission of purified virions nor a direct association between foregut retention and transmission of the virus was determined [20].

In this study, we have contributed a new understanding of the transmission of criniviruses with the first demonstration of an association between foregut retention and transmission of a crinivirus (LCV) by two *B. tabaci* cryptic vector species, i.e., *B. tabaci* NW and MEAM1. This is discernably different from LIYV, where foregut retention and transmission are observed with NW vectors but not with MEAM1 non-vectors [10]. Support for this conclusion was derived from the results presented in Table 1, showing that GH-LCV acquired from infected lettuce plants was retained in the foreguts of the two vector species and transmitted. These transmission results concurred with previous reports that indicated plant-to-plant transmission efficiency of LCV by groups of multiple NW or MEAM1 vectors (similar to the experiments performed in our study (Table 1)) was relatively comparable [5,24]. 

Another contribution made by the present study is the demonstration that GH-LCV virions purified from infected *C. murale* plants using 2% and/or 4% (*v*/*v*) TX-100 were retained in the foreguts of NW and MEAM1 vectors and transmitted to plants following inoculation feeding (Figure 2B and Figure 3C). These findings are consistent with the hypothesis that, as with LIYV, the NCSP transmission of LCV is also facilitated by a “capsid” strategy. Thus, LCV is the second crinivirus (after LIYV) for which the retention of ingested purified virions in a vector’s foregut has been shown to be associated with virus transmission.

The apparent lack of association between the foregut retention of GH-LCV virions (purified using 2% (*v*/*v)* TX-100) and virus transmission by MEAM1 vectors (Figure 3B) was perplexing and may be tied to the sporadic appearance of a viscous supernatant after the first ultracentrifugation step in the virion purification procedure. The causes (and identity) of the viscous supernatant are unknown but could be associated with the incomplete solubilization of membrane-associated constituents. Virion infectivity or transmissibility is known to be affected by the quality or stability of the virion preparation [25,26,27]. Increasing the TX-100 concentration up to 5% (*v*/*v*) in the purification procedure of tobacco mosaic virus and plum pox virus has been found to improve the solubility of virus from cellular membrane constituents, to reduce the aggregation of virions, and to increase the virion yield and infectivity [25,26,28]. In our experiments with LCV, doubling the TX-100 concentration from 2% to 4% (*v*/*v*) in the virion extraction buffer, resulted in transmission by MEAM1 vectors (using 100 vectors per target plant) (Figure 3C), similar to that observed in VRT assays performed using NW vectors (Figure 2B). The use of 4% (*v*/*v*) TX-100 improved the average virus yield (expressed as nanogram of virus per gram fresh weight of leaf tissue) based on the data from five independent virion preparations (Table S1). An increase in virus yield, in turn, increased the average virion concentration used in the VRT assays (Table S1), although we do not know the minimum virion concentration(s) that would support LCV transmission by whitefly vectors via membrane feeding. For LIYV, the minimum virion concentration that supports transmission by NW vectors is 0.1 ng µL^−1^ or higher [29]. 

Whatever the effects of 2% TX-100 on virions, it is clear that the same effects could also be present in 2% TX-100 purified LCV virions subjected to VRT assays using NW vectors. Nevertheless, the 2% TX-100 purified virions were consistently retained in the foreguts of, and transmitted by, the NW vectors. In contrast, while retention in the foreguts of MEAM1 vectors was also consistently observed with 4%-TX-100 purified virions, only a very weak correlation was observed in the association of foregut retention and virus transmission. This is seen in Figure 3C, where plants tested positive for LCV transmission (the red-filled symbols) are quite evenly distributed above and below the mean percentage of foreguts with fluorescent signals. Thus, considered together, all the evidence provides a compelling argument to suggest that there is an intrinsic difference between NW and MEAM1 vectors in their retention and transmission of purified LCV virions. Our previous study showed that the inoculation of foregut bound LIYV virions could be influenced by the dietary pH within the foregut environment [19]. Thus, it is possible that dissimilarities between the foregut environment of NW and MEAM1 vectors, as well as other intrinsic differences such as the nature of their virion-binding sites could be impinging on the foregut retention and transmission mechanism of the virus.

## Figures and Tables

**Table 1:** Foregut retention and transmission of greenhouse (GH)-maintained LCV by *B. tabaci* vectors.

^1^ EXP	*B. tabaci* NW		EXP	*B. tabaci* MEAM1	
	^2^ Retention	^3^ Transmission		Retention	Transmission
1	5.7 ± 1.9	2/4	1	26.4 ± 3.0	3/6
2	23.8 ± 4.4	4/6	2	27.5 ± 4.37	4/6
3	18.2 ± 4.8	3/4	3	19.6 ± 2.74	4/5

^1^ After feeding on a virus infected plant for an overnight AAP, whiteflies were randomly sampled for VRT assays. In Experiment 1, sampled whiteflies were used directly for either retention or transmission assays. Four and six independent samplings of NW and MEAM1 vectors, respectively, were made for these assays. For Experiments 2 and 3, sampled whiteflies were pooled; each pooled sample was re-distributed into two groups of approximately equal numbers. One group was subjected to retention assays, while the other was used for transmission assays. Four to six independent samplings were made in both experiments. ^2^ Average percentage (%) ± standard error (SE) of whiteflies with fluorescent signals in their foreguts determined using four to six samplings for each experiment. ^3^ Transmission scores are presented as the number of plants infected over the total number of target plants tested.

## Data Availability

Data supporting the reported results are available from the corresponding author upon reasonable request.

## Supplementary Materials

The following are available online at https://www.mdpi.com/article/10.3390/v13081578/s1, Table S1: Comparison of the yield and concentration of GH-LCV virions subjected to the VRT assays performed in this study.
