# Supplementary material for: Nuances of Whitefly Vector–Crinivirus Interactions Revealed in the Foregut Retention and Transmission of Lettuce Chlorosis Virus by Two Bemisia tabaci Cryptic Species
Source: Viruses. 2021 Aug 10;13(8):1578. doi: 10.3390/v13081578 (PMC8402701; doi:10.3390/v13081578)
Supplement: Supplementary file 1 [file viruses-13-01578-s001.zip › viruses-1241632-si final.pdf]

Table S1: Comparison of the yield and concentration of GH-LCV virions subjected to the VRT assays in this study.

% TX-100[1] (v/v)	Virus yield (ng/g tissue[2]) Experiment 1	2	3	4	5	Ave. yield	Ave. concentration (ng/μl^{-1})
2	421.2	157.5	654.1	196.6	605.5	406.9	299.3
4	984	163	618.2	992.5	1215.5	794.6	829.4

[1]Percent volume of Triton™ X-100 used in the virion extraction buffer.

[2]GH-LCV infected *C. murale* plants harvested between 7-12 weeks post-transmission by whitefly vectors.
